# Microbiologically active Mannich bases derived from 1,2,4-triazoles. The effect of C-5 substituent on antibacterial activity

**DOI:** 10.1007/s00044-012-0248-y

**Published:** 2012-09-29

**Authors:** Tomasz Plech, Monika Wujec, Magdalena Majewska, Urszula Kosikowska, Anna Malm

**Affiliations:** 1 Department of Organic Chemistry, Faculty of Pharmacy, Medical University, Chodzki 4A, 20-093 Lublin, Poland; 2 Department of Pharmaceutical Microbiology, Faculty of Pharmacy, Medical University, Chodzki 1, 20-093 Lublin, Poland

**Keywords:** Aminomethylation, Five-membered ring, Opportunistic bacteria, MRSA

## Abstract

Our research proved that chemical character of the C-5 substituent significantly determines the antibacterial activity of the Mannich bases derived from 4,5-disubstituted 1,2,4-triazole-3-thiones. This activity was considerably increased by an introduction of a chlorine atom to the phenyl ring. The obtained compounds were particularly active against opportunistic bacteria (*Bacillus subtilis* and *Bacillus* *cereus*). The antibacterial activity of some Mannich bases was similar or higher than the activity of commonly used antibiotics such as ampicillin and cefuroxime.

## Introduction

Excessive and uncontrolled intake of antibiotics resulted in a selection of bacterial strains resistant to commonly used drugs. Recently, the world has been focused on the appearance of the so-called super resistant NDM-1 gene (Yong *et al*., 2009; Rolain *et al*., 2010) which spreads via DNA segments called plasmids. In the view of growing bacterial drug-resistance, the search of chemical substances which can efficiently treat infections caused by this type of bacteria seems to be necessary.

The Mannich reaction is known to be very useful for the synthesis of antibacterial compounds. This reaction makes it possible to introduce amine fragment into the different chemical scaffolds which can increase the affinity of the obtained molecule toward appropriate molecular target. 1,2,4-Triazole-3-thione derivatives known for their antibacterial activity (Turan-Zitouni *et al*., 2005; Eswaran *et al*., 2009; Shafiee *et al*., 2002) were used by many researchers as substrates for the Mannich reaction. The obtained aminomethyl derivatives included both compounds which acted stronger than their N2-unsubstituted predecessors (Isloor *et al*., 2009; Ashok *et al*., 2007; Bayrak *et al*., 2009a), as well as significantly less active compounds (Bayrak *et al*., 2009b; Almajan *et al*., 2009). In our previous studies we proved that the presence of the 4-bromophenyl moiety in the N-4 position benefited the antibacterial activity of 4,5-disubstituted 1,2,4-triazole-3-thione derivatives (Plech *et al*., 2011a, b). Further research also indicated that the activity of this type of Mannich bases decreases with the increased volume of substituent in the N2 position (Plech *et al*., 2011b). The goal of current research was to analyze the impact of the substituent in the C-5 position on the antibacterial activity of obtained compounds. First of all, it has been decided to examine if, and to what degree, the strength of the new derivatives’ activity changes after introducing a chlorine atom to the phenyl ring. Also, the disparities in the activity of appropriate *ortho*-, *meta*-, and *para*- derivatives were analyzed.

## Results and discussion

### Chemistry

**Scheme 1:**
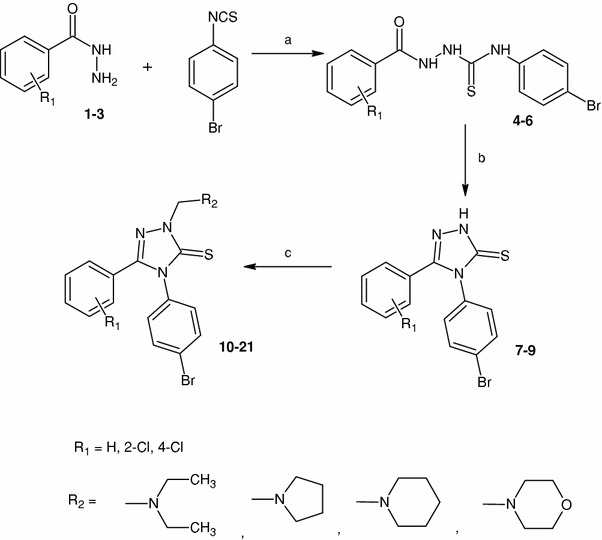
Synthetic route to target compounds **10**–**21**. Reagents and conditions: *a* EtOH, reflux, 5 min; *b* 2 % NaOH, reflux, 2 h; *c* HCHO, amine, EtOH, 30 min

Scheme 1 shows subsequent stages of the synthesis. The substrates for the syntheses included commercially available hydrazides (**1**–**3**). Appropriate thiosemicarbazide derivatives (**4**–**6**) were obtained from the reaction of the hydrazides (**1**–**3**) with 4-bromophenyl isothiocyanate using the method described earlier (Plech *et al*., 2011a). The reaction carried out in the anhydrous ethanol medium lasted 5 min. Spectral and physicochemical properties of the derivatives **4**–**6** were given elsewhere (Li *et al*., 2001; Oruç *et al*., 2004). The cyclization of compounds **4**–**6** in the presence of sodium hydroxide resulted in the formation of 4-(4-bromophenyl)-5-substituted-2,4-dihydro-3*H*-1,2,4-triazole-3-thiones (**7**–**9**). Generally, the 1,2,4-triazole-3-thione derivatives may exist in two tautomeric forms: thione (C=S) and thiole (C–SH). Our earlier studies showed that the thione tautomer is energetically favored (Wujec *et al*., 2007). The IR spectra of compounds **7**–**9** showed the absorption bands at 3,437–3,411 cm^−1^ and 1,331–1,328 cm^−1^, indicating the presence of NH and C=S groups, respectively. In the ^1^H-NMR spectra, NH proton resonated as a singlet at ~14 ppm. Crystallographic data (unpublished results) also confirm the existence of the mentioned compounds as the C=S tautomers.

The Mannich reaction was carried out in mild conditions; it was quick (30 min) and efficient (yields: 76–87 %). The structure and purity of the products (**10**–**21**) was confirmed using ^1^H-NMR, ^13^C-NMR (for compound **20**), and IR spectra as well as elemental analysis. The ^1^H-NMR spectra showed characteristic signals which indicated the presence of aminomethyl fragment. Two protons of the N2–CH_2_– group resonated as a singlet in the range of 5.22–5.34 ppm, while the signals of the amine residues were visible at 1.20–3.76 ppm. In addition to this, peaks characteristic for *para*-substituted phenyl rings were visible in the area typical for aromatic protons. The IR spectra also confirmed the suggested structure of the Mannich bases (**10**–**21**).

### Antibacterial screening

The antibacterial activity of compounds **10**–**21** was determined for Gram-positive and Gram-negative bacteria. The growth of Gram-negative bacteria (*Escherichia coli* ATCC 25922, *Klebsiella pneumoniae* ATCC 13883, *Proteus mirabilis* ATCC 12453, and *Pseudomonas* *aeruginosa* ATCC 9027) was not inhibited by any of the compounds. Therefore, Table 1 shows the Mannich bases activity only for five investigated Gram-positive bacterial strains. The activity toward the pathogenic *Staphylococcus aureus* strains was moderate. Minimum concentrations which inhibited the growth of *S. aureus* ATCC 25923 ranged to 31.25 μg ml^−1^ (**15**, **18**, **19**), and the most active toward methicillin-resistant (MRSA) strain were derivatives with diethylaminomethyl (**18**) and pyrrolidinylmethyl (**19**) substituents. In both cases, the MIC values equaled 62.5 μg ml^−1^. Opportunistic (relatively pathogenic) bacteria was by far more sensitive to the newly obtained compounds. In the case of *Bacillus cereus* ATCC 10876, the activity of three derivatives (**14**, **15**, **21**) was similar to the activity of ampicillin, and the activity of another two derivatives (**18**, **19**) was twice as strong. Moreover, the antibacterial activity of the compound with the N2-pyrrolidinylmethyl fragment (**15**) toward *Bacillus subtilis* ATCC 6633 was as strong as cefuroxime’s; as far as *Micrococcus luteus* ATCC 10240 is concerned, the most active compound was the derivative of 4-(4-bromophenyl)-5-(4-chlorophenyl)-2,4-dihydro-3*H*-1,2,4-triazole-3-thione with pyrrolidinylmethyl substituent (**19**, MIC = 7.81 μg ml^−1^). Based on the MBC/MIC ratio it might be concluded that the obtained compounds do show bactericidal (MBC/MIC ≤4) or bacteriostatic (MBC/MIC >4) activity. This may suggest a presence of more than one mechanism of action for these derivatives.

**Table 1:** In vitro antibacterial screening of compounds **10**–**25** (MICs and MBCs are given in μg ml^−1^)

Compounds	*S. aureus* ATCC 25923		*S. aureus* (MRSA)		*S. epidermidis* ATCC 12228		*B. subtilis* ATCC 6633		*B. cereus* ATCC 10876		*M. luteus* ATCC 10240	
	MIC	MBC	MIC	MBC	MIC	MBC	MIC	MBC	MIC	MBC	MIC	MBC
**10**	1,000	*	1,000	*	1,000	*	1,000	*	1,000	*	1,000	*
**11**	1,000	*	1,000	*	1,000	*	500	1,000	1,000	*	500	1,000
**12**	1,000	*	1,000	*	1,000	*	1,000	*	1,000	*	500	1,000
**13**	1,000	*	1,000	*	1,000	*	1,000	*	1,000	*	250	1,000
**14**	62.5	*	125	*	62.5	*	31.25	62.5	62.5	*	62.5	250
**15**	31.25	500	125	500	31.25	500	15.63	125	62.5	*	62.5	1,000
**16**	500	*	500	*	1,000	*	250	*	1,000	*	500	*
**17**	125	500	250	500	125	500	125	250	125	*	62.5	250
**18**	31.25	250	62.5	250	31.25	250	31.25	500	31.25	250	15.63	62.5
**19**	31.25	500	62.5	1,000	31.25	1,000	62.5	125	31.25	*	7.81	250
**20**	1,000	*	1,000	*	1,000	*	1,000	*	1,000	*	1,000	*
**21**	62.5	1,000	125	*	62.5	1,000	125	125	62.5	*	62.5	*
**22** ^a^	31.25	*	–	–	62.5	*	62.5	500	62.5	*	31.25	500
**23** ^b^	31.25	*	–	–	250	*	62.5	500	62.5	*	62.5	*
**24** ^a^	31.25	*	–	–	62.5	*	62.5	1,000	62.5	1,000	31.25	*
**25** ^a^	31.25	*	–	–	125	*	62.5	1,000	62.5	*	31.25	500
Ampicillin	–	–	–	–	–	–	–	–	62.5	–	–	–
Cefuroxime	0.49	–	–	–	0.24	–	15.63	–	31.25	–	0.98	–
Vancomycin	–	–	0.98	3.91	–	–	–	–	–	–	–	–

*–* not determined, * MIC or MBC values higher than 1,000 μg ml^−1^

^a^Data derived from Plech *et al*. (2011b)

^b^Data derived from Plech *et al*. (2011a)

**Fig. 1:**
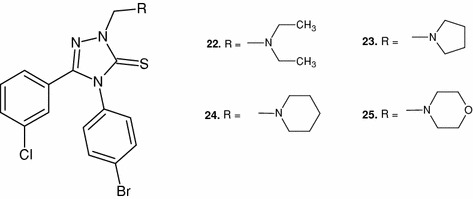
Chemical structures of compounds **22**–**25**

In order to analyze the impact of the type of substituent in the C-5 position on the antibacterial activity, derivatives including phenyl (**10**–**13**), 2-chlorophenyl (**14**–**17**), and 4-chlorophenyl (**18**–**21**) rings were obtained. In order to ensure more comprehensive analysis, the discussion also considered the compounds with 3-chlorophenyl fragment (**22**–**25**) (Fig. 1)—synthesized and described by us recently (Plech *et al*., 2011a, b). Regardless of the type of aminomethyl substituent in the N2 position, none of the C5-phenyl derivatives showed antibacterial activity which would be worth noticing. The activity of the obtained Mannich bases was significantly increased only after an electron-withdrawing chlorine atom had been introduced to the phenyl ring. Interesting conclusions can be drawn when comparing the activity of appropriate *ortho*-, *meta*-, and *para*- analogs. Balanced activity toward all analyzed Gram-positive bacteria was characteristic for compounds with 3-chlorophenyl fragment, regardless of the type of substituent in the N2 position. While the activity of *ortho*- and *para*- analogs depended largely on the type of aminomethyl fragment. It is worth mentioning that compounds with pentatomic (apart from hydrogen atoms) amine substituent in the N2 position (pyrrolidine and diethylamine) were microbiologically more active than appropriate compounds with six-membered substituents (piperidine and morpholine). Also, the disparity between the activities of piperidinyl and morpholinyl derivatives shows that the oxygen atom in the morpholine molecule is important for the binding with a potential molecular target. This is probably caused by the fact that the oxygen atom can participate in the formation of hydrogen bonds in the drug-target site.

## Conclusions

Our research showed that chemical character of the C-5 substituent significantly determines the antibacterial activity of the N2-aminomethyl derivatives of the 1,2,4-triazole. This activity can be considerably increased by an introduction of an electron-withdrawing chlorine atom to the phenyl ring in the C-5 position. In addition to this, the number of atoms which form the aminomethyl substituent seems to be important. The activity of the obtained Mannich bases was particularly strong toward opportunistic bacteria. The antibacterial activity of some compounds was similar or higher than the activity of commonly used antibiotics such as ampicillin and cefuroxime.

## Experimental

### General comments

All reagents and solvents were purchased from Alfa Aesar (Ward Hill, USA) and Merck Co. (Darmstadt, Germany). Melting points were determined using Fisher-Johns apparatus (Fisher Scientific, Schwerte, Germany) and are uncorrected. The ^1^H-NMR and ^13^C-NMR spectra were recorded on a Bruker Avance spectrometer (Bruker BioSpin GmbH, Rheinstetten, Germany) using TMS as an internal standard. The IR spectra (KBr) were obtained on a Perkin-Elmer 1725X FTIR spectrophotometer. Elemental analyses were performed on an AMZ 851 CHX analyzer (PG, Gdańsk, Poland) and the results were within ±0.2 % of the theoretical value. All the compounds were purified by flash chromatography (PuriFlash 430*evo*, Interchim, USA).

### Synthesis of thiosemicarbazide derivatives (**4**–**6**)

Three derivatives of thiosemicarbazide: *1*-*benzoyl*-*4*-*(4*-*bromophenyl)thiosemicarbazide* (**4**), *4*-*(4*-*bromophenyl)*-*1*-*[(2*-*chlorophenyl)carbonyl]thiosemicarbazide* (**5**), and *4*-*(4*-*bromophenyl)*-*1*-*[(4*-*chlorophenyl)carbonyl]thiosemicarbazide* (***6***) were synthesized according to the procedure described earlier (Plech *et al*., 2011a). Their spectral and physicochemical properties were consistent with (Li *et al*., 2001; Oruç *et al*., 2004).

### Synthesis of 1,2,4-triazole derivatives **(7**–**9)**

Appropriate thiosemicarbazides (**4**–**6**) were dissolved in 2 % solution of NaOH. Next, the resulting solution was heated under reflux for 2 h. After cooling, the reaction mixture was neutralized with HCl. The precipitated product was filtered off, washed with distilled water, and recrystallized from EtOH.

#### *4*-*(4*-*Bromophenyl)*-*5*-*phenyl*-*2,4*-*dihydro*-*3H*-*1,2,4*-*triazole*-*3*-*thione* (**7**)

Yield: 87 %, CAS Registry Number: 162221-97-8.

#### *4*-*(4*-*Bromophenyl)*-*5*-*(2*-*chlorophenyl)*-*2,4*-*dihydro*-*3H*-*1,2,4*-*triazole*-*3*-*thione* (**8**)

Yield: 83 %, m.p. 282–284 °C, ^1^H-NMR (250 MHz) (DMSO-*d*_6_) *δ* (ppm): 7.08–7.76 (m, 8H, Ar–H), 14.03 (s, 1H, NH, exch. D_2_O). IR (KBr, ν, cm^−1^): 3411, 3045, 1531, 1331. *Anal.* Calc. for C_14_H_9_BrClN_3_S (%): C 45.86, H 2.47, N 11.46. Found: C 45.99, H 2.35, N 11.43.

#### *4*-*(4*-*Bromophenyl)*-*5*-*(4*-*chlorophenyl)*-*2,4*-*dihydro*-*3H*-*1,2,4*-*triazole*-*3*-*thione* (**9**)

Yield: 82 %, CAS Registry Number: 537017-82-6.

### General procedure for the synthesis of Mannich bases **(10**–**21)**

10 mmol of the 1,2,4-triazole derivative (**7**–**9**) was dissolved (with heating) in 20 ml of anhydrous ethanol and then equimolar amounts of appropriate secondary amine (diethylamine, pyrrolidine, piperidine, and morpholine) and formaldehyde solution (37 %) were added. The obtained mixture was stirred at room temperature for 30 min. Next, 5 ml of distilled water was added, the precipitate was filtered off, washed with distilled water, and recrystallized from ethanol.

#### *4*-*(4*-*Bromophenyl)*-*2*-*[(diethylamino)methyl]*-*5*-*phenyl*-*2,4*-*dihydro*-*3H*-*1,2,4*-*triazole*-*3*-*thione* (**10**)

Yield: 78 %, m.p. 118–120 °C, ^1^H-NMR (250 MHz) (CDCl_3_) *δ* (ppm): 1.20 (t, 6H, 2 × CH_3_, *J* = 7.17 Hz), 2.90 (q, 4H, 2 × CH_2_, *J* = 7.18 Hz), 5.32 (s, 2H, CH_2_), 7.18 (d, 2H, Ar–H, *J* = 8.69 Hz), 7.25–7.34 (m, 5H, Ar–H), 7.61 (d, 2H, Ar–H, *J* = 8.70 Hz). IR (KBr, ν, cm^−1^): 3065, 2931, 2796, 1612, 1520, 1331, 799. *Anal.* Calc. for C_19_H_21_BrN_4_S (%): C 54.68, H 5.07, N 13.42. Found: C 54.60, H 5.02, N 13.53.

#### *4*-*(4*-*Bromophenyl)*-*5*-*phenyl*-*2*-*(pyrrolidin*-*1*-*ylmethyl)*-*2,4*-*dihydro*-*3H*-*1,2,4*-*triazole*-*3*-*thione* (**11**)

Yield: 82 %, m.p. 142–143 °C, ^1^H-NMR (250 MHz) (CDCl_3_) *δ* (ppm): 1.75–1.83 (m, 4H, 2 × CH_2_), 2.99 (t, 4H, 2 × CH_2_, *J* = 6.43 Hz), 5.34 (s, 2H, CH_2_), 7.19 (d, 2H, Ar–H, *J* = 8.86 Hz), 7.25–7.33 (m, 5H, Ar–H), 7.61 (d, 2H, Ar–H, *J* = 8.84 Hz). IR (KBr, ν, cm^−1^): 3084, 3008, 2915, 2868, 1584, 1513, 1323, 806. *Anal.* Calc. for C_19_H_19_BrN_4_S (%): C 54.94, H 4.61, N 13.49. Found: C 55.05, H 4.50, 13.50.

#### *4*-*(4*-*Bromophenyl)*-*5*-*phenyl*-*2*-*(piperidin*-*1*-*ylmethyl)*-*2,4*-*dihydro*-*3H*-*1,2,4*-*triazole*-*3*-*thione* (**12**)

Yield: 77 %, m.p. 122–123 °C, ^1^H-NMR (250 MHz) (CDCl_3_) *δ* (ppm): 1.44–1.68 (m, 6H, 3 × CH_2_), 2.87 (t, 4H, 2 × CH_2_, *J* = 5.40 Hz), 5.25 (s, 2H, CH_2_), 7.19 (d, 2H, Ar–H, *J* = 8.90 Hz), 7.24–7.35 (m, 5H, Ar–H), 7.61 (d, 2H, Ar–H, *J* = 8.90 Hz). IR (KBr, ν, cm^−1^): 3110, 2918, 2785, 1603, 1519, 1342, 808. *Anal.* Calc. for C_20_H_21_BrN_4_S (%): C 55.94, H 4.93, N 13.05. Found: C 56.00, H 4.90, N 13.17.

#### *4*-*(4*-*Bromophenyl)*-*2*-*(morpholin*-*4*-*ylmethyl)*-*5*-*phenyl*-*2,4*-*dihydro*-*3H*-*1,2,4*-*triazole*-*3*-*thione* (**13**)

Yield: 83 %, m.p. 146–147 °C, ^1^H-NMR (250 MHz) (CDCl_3_) *δ* (ppm): 2.95 (t, 4H, 2 × CH_2_, *J* = 4.26 Hz), 3.76 (t, 4H, 2 × CH_2_, *J* = 4.26 Hz), 5.26 (s, 2H, CH_2_), 7.18 (d, 2H, Ar–H, *J* = 8.80 Hz), 7.24–7.35 (m, 5H, Ar–H), 7.62 (d, 2H, Ar–H, *J* = 8.81 Hz). IR (KBr, ν, cm^−1^): 3074, 3021, 2961, 2831, 1574, 1512, 1328, 786. *Anal.* Calc. for C_19_H_19_BrN_4_OS (%): C 52.90, H 4.44, N 12.99. Found: C 52.98, H 4.56, N 13.05.

#### *4*-*(4*-*Bromophenyl)*-*5*-*(2*-*chlorophenyl)*-*2*-*[(diethylamino)methyl]*-*2,4*-*dihydro*-*3H*-*1,2,4*-*triazole*-*3*-*thione* (**14**)

Yield: 79 %, m.p. 172–173 °C, ^1^H-NMR (250 MHz) (CDCl_3_) *δ* (ppm): 1.20 (t, 6H, 2 × CH_3_, *J* = 7.55 Hz), 2.90 (q, 4H, 2 × CH_2_, *J* = 7.55 Hz), 5.30 (s, 2H, CH_2_), 7.17 (d, 2H, Ar–H, *J* = 8.89 Hz), 7.22–7.32 (m, 4H, Ar–H), 7.62 (d, 2H, Ar–H, *J* = 8.90 Hz). IR (KBr, ν, cm^−1^): 3030, 2986, 2832, 1603, 1541, 1341, 813. *Anal.* Calc. for C_19_H_20_BrClN_4_S (%): C 50.51, H 4.46, N 12.40. Found: C 50.41, H 4.38, N 12.29.

#### *4*-*(4*-*Bromophenyl)*-*5*-*(2*-*chlorophenyl)*-*2*-*(pyrrolidin*-*1*-*ylmethyl)*-*2,4*-*dihydro*-*3H*-*1,2,4*-*triazole*-*3*-*thione* (**15**)

Yield: 84 %, m.p. 143–145 °C, ^1^H-NMR (250 MHz) (CDCl_3_) *δ* (ppm): 1.76–1.83 (m, 4H, 2 × CH_2_), 2.96 (t, 4H, 2 × CH_2_, *J* = 6.40 Hz), 5.32 (s, 2H, CH_2_), 7.17 (d, 2H, Ar–H, *J* = 8.75 Hz), 7.22–7.30 (m, 4H, Ar–H), 7.63 (d, 2H, Ar–H, *J* = 8.75 Hz). IR (KBr, ν, cm^−1^): 3099, 2956, 2825, 1589, 1530, 1327, 802. *Anal.* Calc. for C_19_H_18_BrClN_4_S (%): C 50.73, H 4.03, N 12.46. Found: C 50.66, H 4.12, N 12.45.

#### *4*-*(4*-*Bromophenyl)*-*5*-*(2*-*chlorophenyl)*-*2*-*(piperidin*-*1*-*ylmethyl)*-*2,4*-*dihydro*-*3H*-*1,2,4*-*triazole*-*3*-*thione* (**16**)

Yield: 80 %, m.p. 180–181 °C, ^1^H-NMR (250 MHz) (CDCl_3_) *δ* (ppm): 1.36–1.69 (m, 6H, 3 × CH_2_), 2.85 (t, 4H, 2 × CH_2_, *J* = 5.40 Hz), 5.22 (s, 2H, CH_2_), 7.18 (d, 2H, Ar–H, *J* = 8.71 Hz), 7.23–7.34 (m, 4H, Ar–H), 7.63 (d, 2H, Ar–H, *J* = 8.70 Hz). IR (KBr, ν, cm^−1^): 3062, 2985, 2800, 1594, 1526, 1342, 784. *Anal.* Calc. for C_20_H_20_BrClN_4_S (%): C 51.79, H 4.35, N 12.08. Found: C 51.90, H 4.35, N 12.00.

#### *4*-*(4*-*Bromophenyl)*-*5*-*(2*-*chlorophenyl)*-*2*-*(morpholin*-*4*-*ylmethyl)*-*2,4*-*dihydro*-*3H*-*1,2,4*-*triazole*-*3*-*thione* (**17**)

Yield: 76 %, m.p. 174–175 °C, ^1^H-NMR (250 MHz) (CDCl_3_) *δ* (ppm): 2.91 (t, 4H, 2 × CH_2_, *J* = 4.75 Hz), 3.72 (t, 4H, 2 × CH_2_, *J* = 4.75 Hz), 5.23 (s, 2H, CH_2_), 7.17 (d, 2H, Ar–H, *J* = 8.81 Hz), 7.23–7.34 (m, 4H, Ar–H), 7.64 (d, 2H, Ar–H, *J* = 8.81 Hz). IR (KBr, ν, cm^−1^): 3037, 2903, 2785, 1600, 1521, 1328, 806. *Anal.* Calc. for C_19_H_18_BrClN_4_OS (%): C 48.99, H 3.90, N 12.03. Found: C 49.11, H 3.84, N 12.17.

#### *4*-*(4*-*Bromophenyl)*-*5*-*(4*-*chlorophenyl)*-*2*-*[(diethylamino)methyl]*-*2,4*-*dihydro*-*3H*-*1,2,4*-*triazole*-*3*-*thione* (**18**)

Yield: 82 %, m.p. 175–176 °C, ^1^H-NMR (250 MHz) (CDCl_3_) *δ* (ppm): 1.20 (t, 6H, 2 × CH_3_, *J* = 7.24 Hz), 2.90 (q, 4H, 2 × CH_2_, *J* = 7.24 Hz), 5.30 (s, 2H, CH_2_), 7.17 (d, 2H, Ar–H, *J* = 8.63 Hz), 7.22–7.33 (m, 4H, Ar–H), 7.62 (d, 2H, Ar–H, *J* = 8.63 Hz). IR (KBr, ν, cm^−1^): 3088, 3009, 2917, 2826, 1589, 1526, 1319, 778. *Anal.* Calc. for C_19_H_20_BrClN_4_S (%): C 50.51, H 4.46, N 12.40. Found: C 50.43, H 4.52, N 12.41.

#### *4*-*(4*-*Bromophenyl)*-*5*-*(4*-*chlorophenyl)*-*2*-*(pyrrolidin*-*1*-*ylmethyl)*-*2,4*-*dihydro*-*3H*-*1,2,4*-*triazole*-*3*-*thione* (**19**)

Yield: 87 %, m.p. 143–144 °C, ^1^H-NMR (250 MHz) (CDCl_3_) *δ* (ppm): 1.76–1.84 (m, 4H, 2 × CH_2_), 2.97 (t, 4H, 2 × CH_2_, *J* = 6.10 Hz), 5.32 (s, 2H, CH_2_), 7.18 (d, 2H, Ar–H, *J* = 8.76 Hz), 7.23–7.34 (m, 4H, Ar–H), 7.64 (d, 2H, Ar–H, *J* = 8.76 Hz). IR (KBr, ν, cm^−1^): 3040, 2921, 2787, 1609, 1541, 1333, 783. *Anal.* Calc. for C_19_H_18_BrClN_4_S (%): C 50.73, H 4.03, N 12.46. Found: C 50.62, H 3.92, N 12.52.

#### *4*-*(4*-*Bromophenyl)*-*5*-*(4*-*chlorophenyl)*-*2*-*(piperidin*-*1*-*ylmethyl)*-*2,4*-*dihydro*-*3H*-*1,2,4*-*triazole*-*3*-*thione* (**20**)

Yield: 76 %, m.p. 180–182 °C, ^1^H-NMR (250 MHz) (CDCl_3_) *δ* (ppm): 1.36–1.68 (m, 6H, 3 × CH_2_), 2.85 (t, 4H, 2 × CH_2_, *J* = 5.62 Hz), 5.22 (s, 2H, CH_2_), 7.18 (d, 2H, Ar–H, *J* = 8.74 Hz), 7.23-7.31 (m, 4H, Ar–H), 7.63 (d, 2H, Ar–H, *J* = 8.72 Hz). ^13^C-NMR (90 MHz) (CDCl_3_) *δ* (ppm): 23.81, 25.91, 51.82, 71.09, 123.64, 124.10, 129.11, 129.87, 130.02, 133.27, 134.45, 137.27, 148.18, 170.64. IR (KBr, ν, cm^−1^): 3085, 2882, 2790, 1600, 1531, 1323, 809. *Anal.* Calc. for C_20_H_20_BrClN_4_S (%): C 51.79, H 4.35, N 12.08. Found: C 51.86, H 4.32, N 12.18.

#### *4*-*(4*-*Bromophenyl)*-*5*-*(4*-*chlorophenyl)*-*2*-*(morpholin*-*4*-*ylmethyl)*-*2,4*-*dihydro*-*3H*-*1,2,4*-*triazole*-*3*-*thione* (**21**)

Yield: 80 %, m.p. 177–178 °C, ^1^H-NMR (250 MHz) (CDCl_3_) *δ* (ppm): 2.91 (t, 4H, 2 × CH_2_, *J* = 4.73 Hz), 3.73 (t, 4H, 2 × CH_2_, *J* = 4.70 Hz), 5.23 (s, 2H, CH_2_), 7.17 (d, 2H, Ar–H, *J* = 8.70 Hz), 7.25–7.34 (m, 4H, Ar–H), 7.64 (d, 2H, Ar–H, *J* = 8.70 Hz). IR (KBr, ν, cm^−1^): 3074, 3033, 2951, 2856, 1603, 1541, 1318, 798. *Anal.* Calc. for C_19_H_18_BrClN_4_OS (%): C 48.99, H 3.90, N 12.03. Found: C 49.10, H 3.97, N 12.00.

### Antibacterial screening

Tested microorganism: *S. aureus* ATCC 25923, *S. aureus* Microbank 14001 (MRSA), *Staphylococcus epidermidis* ATCC 12228, *B. subtilis* ATCC 6633, *B. cereus* ATCC 10876, *M. luteus* ATCC 10240, *E. coli* ATCC 25922, *K. pneumoniae* ATCC 13883, *P. mirabilis* ATCC 12453, and *P. aeruginosa* ATCC 9027. Preliminary antibacterial in vitro potency of the tested compounds was screened using the agar dilution method on the basis of the growth inhibition on the Mueller–Hinton agar to which the tested compounds at concentration 1,000 μg ml^−1^ were added. The plates were poured on the day of testing. 10 μl of each bacterial suspension was put onto Mueller–Hinton agar containing the tested compounds; medium without the compounds was used as a control. The plates were incubated at 37 °C for 18 h. Then the in vitro antibacterial activity of the compounds with inhibitory effect was determined by broth microdilution method. Ampicillin, cefuroxime, and vancomycin were used as control antimicrobial agents. The microbial suspensions were prepared in sterile saline with an optical density of 0.5 McFarland standard—150 × 10^6^ CFU ml^−1^ (CFU—colony forming unit). All stock solutions of the tested compounds were dissolved in DMSO. Mueller–Hinton broth was used with a series of twofold dilutions of the tested substances in the range of final concentrations from 3.91 to 1,000 μg ml^−1^. Minimum inhibitory concentration (MIC) and minimum bactericidal concentration (MBC) are given in μg ml^−1^ (CLSI 2008).
